# Welcome from the new editor-in-chief

**DOI:** 10.1007/s12471-024-01855-x

**Published:** 2024-02-01

**Authors:** Pim van der Harst

**Affiliations:** Department of Cardiology, Division of Heart & Lungs, University Medical Center Utrecht, Utrecht, The Netherlands

I am honoured and pleased to accept the role of Editor-in-Chief of our journal. It is a privilege to continue the exemplary work of my predecessors. Jan Piek, who admirably held this role for seven years, built upon the 30-year legacy of Ernst E. van der Wall. Stepping into the footsteps of these cardiology giants presents a significant challenge, but I am committed to continually renewing and improving the journal to meet future challenges and maintain its vital role in the cardiovascular community, both within the Netherlands and internationally.

Given the strong foundation upon which the NHJ is built, there will be no immediate or radical changes. However, the NHJ will persistently renew and reinvent itself. We will maintain our focus on our base in the Netherlands, disseminating important Dutch data. For instance, the current issue reports on the cardiovascular and bleeding risks in elderly NSTEMI patients in the Netherlands [1], and shares new Dutch experiences of implementing a cardiology-geriatric co-management ward for patients aged 85 and over [2].

Furthermore, the journal will continue to develop its crucial role within the Netherlands Society of Cardiology (NVVC), for example by disseminating insights on applying European guidelines in Dutch healthcare settings. As exemplified in the current issue, this will not withhold us from also reporting data that challenge guidelines [3]. Excellent reviews on timely topics and special issues from our expert authors will enhance our international readership and visibility as the NHJ is one of the few national cardiology journals with an international impact factor.

Looking ahead, increasing the visibility of our authors and the NVVC will be a key focus. We will also invest in attractive and innovative ways of information dissemination.

I eagerly anticipate collaborating with our talented authors, peer reviewers, editorial board, the NVVC, and Bohn Stafleu van Loghum. I am also particularly excited to work with Lieda Meester at the Editorial Office. Her longstanding expertise and guidance are invaluable to the editorial board and the journal’s continued success.
